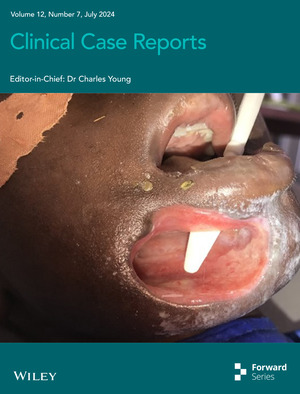# Cover Image

**DOI:** 10.1002/ccr3.9181

**Published:** 2024-07-17

**Authors:** Zilefac Brian Ngokwe, Nokam Kamdem Stephane Gimel, Ntep Ntep David Bienvenue, Ginette Claude Mireille Kalla, Bengondo Messanga Charles

## Abstract

The cover image is based on the Case Report *Cancrum oris and hemiparesis in a young female patient—a case report* by Zilefac Brian Ngokwe et al., https://doi.org/10.1002/ccr3.9111